# Genome-wide identification of *Arabidopsis* long noncoding RNAs in response to the blue light

**DOI:** 10.1038/s41598-020-63187-1

**Published:** 2020-04-10

**Authors:** Zhenfei Sun, Kai Huang, Zujing Han, Pan Wang, Yuda Fang

**Affiliations:** 1 Joint Center for Single Cell Biology, School of Agriculture and Biology, Shanghai Jiao Tong University, Shanghai, 200240 China; 2 National Key Laboratory of Plant Molecular Genetics, CAS Center for Excellence in Molecular Plant Sciences, Institute of Plant Physiology and Ecology, Chinese Academy of Sciences; University of Chinese Academy of Sciences, Shanghai, 200032 China; 3 Beijing igeneCode Biotech CO., Ltd, Beijing, 100096 China

**Keywords:** Light responses, Plant molecular biology

## Abstract

Long non-coding RNAs (lncRNAs) have been shown in animals to play roles in a wide range of biological processes. In plant, light modulates the growth and development as a key external signal. However, little is known about the role of plant lncRNA in response to light. In this study, we sequenced the messenger RNAs (mRNAs), lncRNAs and microRNAs (miRNAs) in *Arabidopsis* seedlings under blue light for 2 h and 8 h. Compared to dark, we identified 4197 mRNAs, 375 miRNAs and 481 lncRNAs, or 5207 mRNAs, 286 miRNAs and 545 lncRNAs of differential expressions under blue light treatments for 2 h or 8 h respectively. Subsequently, a total of 407 competing endogenous RNA (ceRNA) pairs (lncRNA-mRNA-miRNA) were constructed. We identified a blue light-induced lncRNA which plays roles in blue light-directed plant photomorphogenesis and response to mannitol stress by serving as a ceRNA to sequester miR167 in a type of target mimicry. These results revealed previously unknown roles of the lncRNA in blue light signaling and mannitol stress, and provided useful resources of lncRNAs associated with miRNAs in response to blue light.

## Introduction

Long non-coding RNAs (lncRNAs) represent transcripts of more than 200 nucleotides in length without or with little protein-coding potential. Previous studies have shown that lncRNAs are involved in many biological processes, including chromatin structure regulation, stem cell fate determination, tumorgenesis, etc. LncRNAs affect gene expression by acting as cis-acting elements, trans-acting factors, scaffolding components of chromatin-modified protein complexes, or nuclear body maintenance factors^1–3^.

LncRNAs have been extensively studied in animals. In contrast, the number of plant lncRNAs that have been functionally characterized are limited^4,5^. Plant lncRNAs have been found to be involved in gene silencing^6^, flowering time regulation (*COLD ASSISTED INTRONIC NONCODING RNA* (COLDAIR and COOLAIR)^7^;^8^, biotic and abiotic stress responses^9^, male fertility (*LONG-DAY-SPECIFIC MALE-FERTILITY-ASSOCIATED RNA*, LDMAR)^10^, photomorphogenesis^11^, plant disease resistance^12^, and regulation of phosphate assimilation (*INDUCED BY PHOSPHATE STARVATION1*, IPS1/At4)^13^;^14^.

Interestingly, some lncRNAs can serve as signal molecules under different stimulation conditions^15^. In addition, the non-coding IPS1 (INDUCED BY PHOSPHATE STARVATION 1) RNA can serve as a molecular bait, which binds directly to ceRNA to block the role of the targets (Franco-Zorrilla *et al*., 2007), or to transcription factor/regulator to inhibit the related signal transduction. The ceRNA is the RNA component in a complex for transcriptional regulation through RNA-RNA interaction. CeRNAs include some of the protein-coding RNAs, lncRNAs, miRNAs, pseudogene RNAs, and circular RNAs (circRNAs)^16^. The function of the interaction between the lncRNA and its miRNA target was shown in the sequestration of miR399 by plant lncRNA IPS1/At4 to regulate phosphate homeostasis^14^. LncRNA can act as a directing factor: after binding to protein, it can locate the protein complexes on specific DNA sequences, and regulate the transcription of downstream factors. In addition, lncRNAs can serve as scaffold molecules to bind to multiple transcription factors to activate multiple signaling pathways at the same time, and achieve information integration between different signaling pathways^17^.

As one of the most important environmental factors, light regulates plant growth and development in many aspects, including germination, photomorphogenesis, phototropism, flowering, stem and leaf growth, clock regulation, stomatal opening, chloroplast movement and anthocyanin synthesis^18^. Plants use at least four types of photoreceptors to receive spectral signals of different wavelengths, including phytochromes (phys), cryptochromes (crys), phototropins (phots), and ultraviolet B receptors. PhyA to phyE are receptors of red and far red lights^19–22^. Three types of blue light receptors were identified: cryptochrome (including CRY1 and CRY2), phototropin (including PHOT1 and PHOT2) and LOV/F-box/Kelch-repeat protein (including ZTL, LKP2 and FKF1)^21^. The interactions between blue light receptors LKP2 and FKF1 and the transcription factor CDF2 play important roles in blue light signal transduction through ubiquitination-mediated protein degradation^23^. In previous works, we revealed that CDF2 is involved in miRNA transcription and processing^24^, and PIF4, which was known to interact with phyB and CRY1^25,26^, plays a role in miRNA biogenesis to affect red light-directed plant photomorphogenesis^27^.

Here we found that blue light induced the expressions of *Arabidopsis* lncRNAs along with the changed expressions of a group of miRNAs under blue light. By screening the mutants of *lncRNAs*, we identified a blue light-induced lncRNA (NONATHG000085.1) (named BLIL1) which was able to inhibit hypocotyl elongation under blue light. Bioinformatics analysis showed a competitive relationship between BLIL1 and miRNA167. We characterized the ceRNA relationship between BLIL1 and miRNA167 by analyzing the levels of BLIL1, miRNA167 and the transcripts of miRNA167-targeted genes *ARF6/8*, as well as the phenotypes of their mutants under blue light or mannitol treatments. Our results demonstrated a previously unknown role of lncRNA in the blue light signaling and mannitol stress response.

## Results

### High-throughput sequencing of the transcripts from the *Arabidopsis* seedlings upon blue light treatments

**Figure 1:**
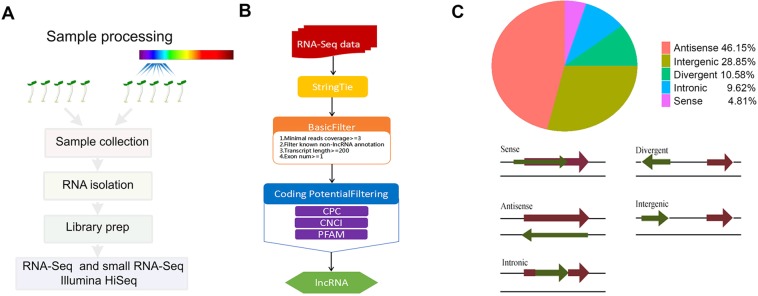
Definition of the lncRNA transcriptome of *Arabidopsis* under blue light. **(A)** Sample processing overview. **(B)** LncRNA identification overview. **(C)** Positional classification of lncRNA loci relative to the nearest protein coding gene.

To annotate the relationships between lncRNAs and miRNAs in *Arabidopsis* under blue light, we performed lncRNA sequencing of three biological replicates, each with three samples using 4 day dark-grown WT seedlings exposed to continuous blue light for 0 h, 2 h and 8 h (Fig. 1A). 1,095,370–1,100,180 M paired-end reads with 100 bp in length for the mRNAs and lncRNAs, and 11,600,936–12,413,799 single-end reads with 50 bp in length for the miRNAs were acquired from nine samples (Table S1 in Supporting Information). All the clean reads were aligned to *Arabidopsis* reference genome assembly (UMD3.1.80) for further analysis. We calculated the correlation coefficient (r^2^) of the sequencing data among the three individual samples of biological replicates in each period based on the FPKM and TPM values, and found that the r^2^ values were 0.8499–0.9958, 0.9774–0.9995 and 0.8955–0.9907 for the mRNAs, lncRNAs and miRNAs, respectively, indicating that the similarities of the mRNA, lncRNA or miRNA biological replicates were sufficiently high (Fig. S1).

### **Identification of lncRNAs in*Arabidopsis*seedlings under blue light**

To annotate lncRNAs, we extracted the total RNA from samples. Non-coding RNAs and mRNAs were enriched by removing the ribosomal RNAs (rRNAs) from the total RNA. Due to the instability of rRNA removing efficiency, we also removed rRNA-contained reads by alignment. We choose Coding Poteintial Calculator (CPC)^28^ and iSeeRNA^29^ which perform well compared to other softwares in both accuracy and efficiency for lncRNA prediction. Cuffmege was used to merge several assemblies together and filter the transfrags that are probably artifacts. A single annotation file was then generated for downstream differential analysis. We obtained the whole parsimonious set of transcripts to detect the novel transcripts from the initial assemblies, and compared the assembled transcripts to the reference annotation. HISAT reference annotation based assembly method was used to reconstruct the transcripts, and background noise was reduced by using FPKM and coverage threshold^30,31^. The novel transcripts were identified by calculating the coding potential of these transcripts (Fig. 1B).

We also calculated the distribution of lncRNA loci induced under blue light treatments for 2 h and 8 h in NONCODE v3.0^32^ with Cuffcompare categories. We found that the majority of lncRNA loci (46.15%) are antisense, while 28.85% are intergenic, 10.58% divergent, 9.62% intronic and 4.81% sense (Fig. 1C).

### Identification of DEmRNAs, DEmiRNAs, and DElncRNAs in *Arabidopsis* seedlings under blue light

**Figure 2:**
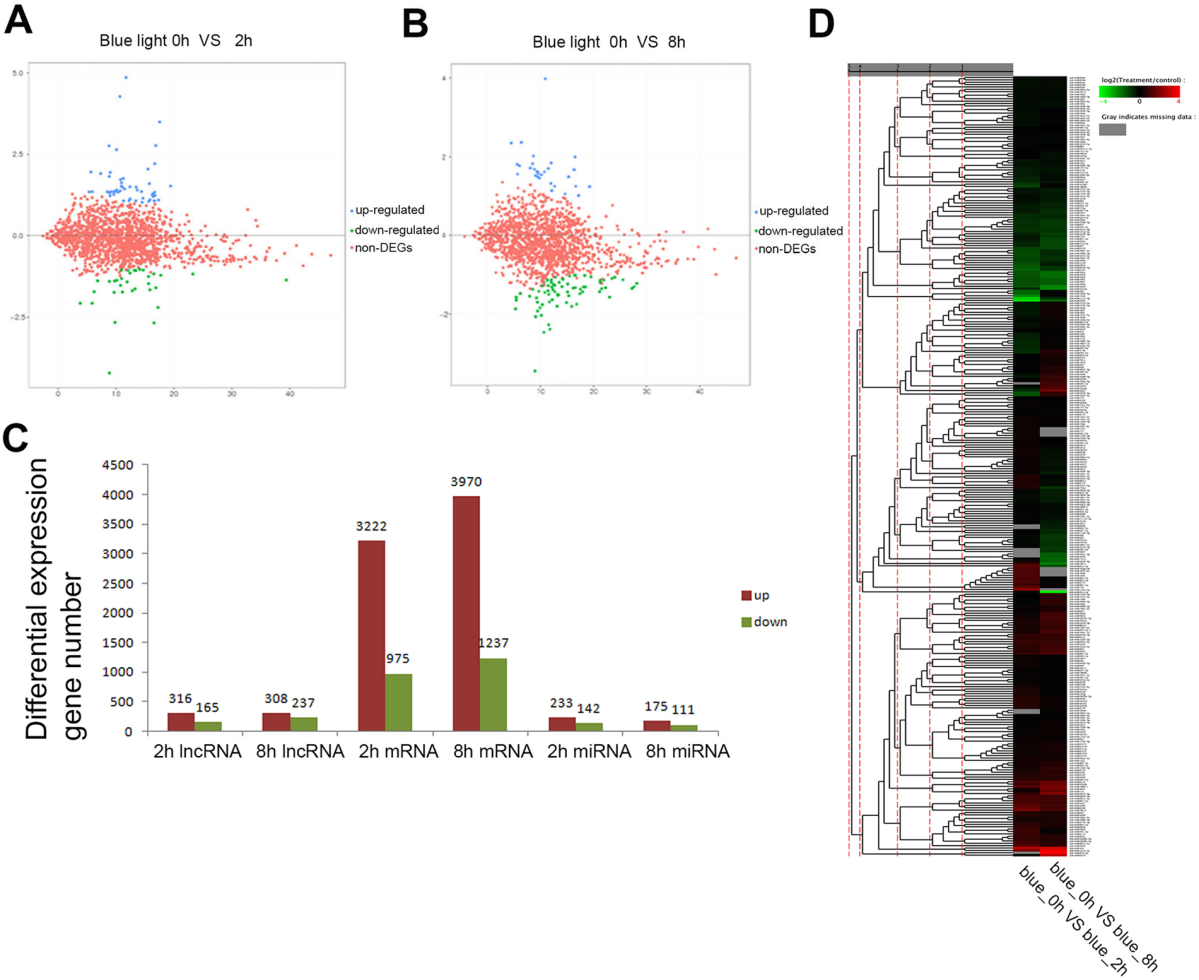
Comparisons of lncRNAs, mRNAs and miRNAs differentially expressed in 4d Col seedlings under blue light. **(A)** Comparison of lncRNAs differentially expressed in 4d Col seedlings between blue light treatments for 0 h and 2 h. **(B)** Comparison of lncRNAs differentially expressed in 4d Col seedlings between blue light treatments for 0 h and 8 h. **(C)** Comparison of miRNAs, lnRNAs and mRNAs differentially expressed in 4d Col seedlings between blue light treatments for 0 h and 2 h, or 0 h and 8 h. **(D)** The statistics of differential expressions of lncRNAs, mRNAs and miRNAs between blue light treatment for 0 h and 2 h, or 0 h and 8 h.

To identify the regulatory networks of mRNAs, lncRNAs and miRNAs under blue light, we analyzed the differentially expressed mRNAs, lncRNAs and miRNAs (DEmRNAs, DElncRNAs, DEmiRNAs). Compared to the dark condition, 481 or 545 DElncRNAs were found in *Arabidopsis* seedlings under blue light treatment for 2 h or 8 h respectively, (Fig. 2A,B), of which 316 or 308 were up-regulated and 165 or 237 down-regulated. In addition, 4197 or 5207 DEmRNAs were identified in *Arabidopsis* seedlings under blue light treatment for 2 h or 8 h respectively, with 3222 or 3970 up-regulated and 975 or 1237 down-regulated more than two fold. In total, 375 or 286 DEmiRNAs were found in *Arabidopsis* seedlings under blue light treatments for 2 h or 8 h respectively, with 233 or 175 up-regulated and 142 or 111 down-regulated for more than two fold (Fig. 2C,D).

**Figure 3:**
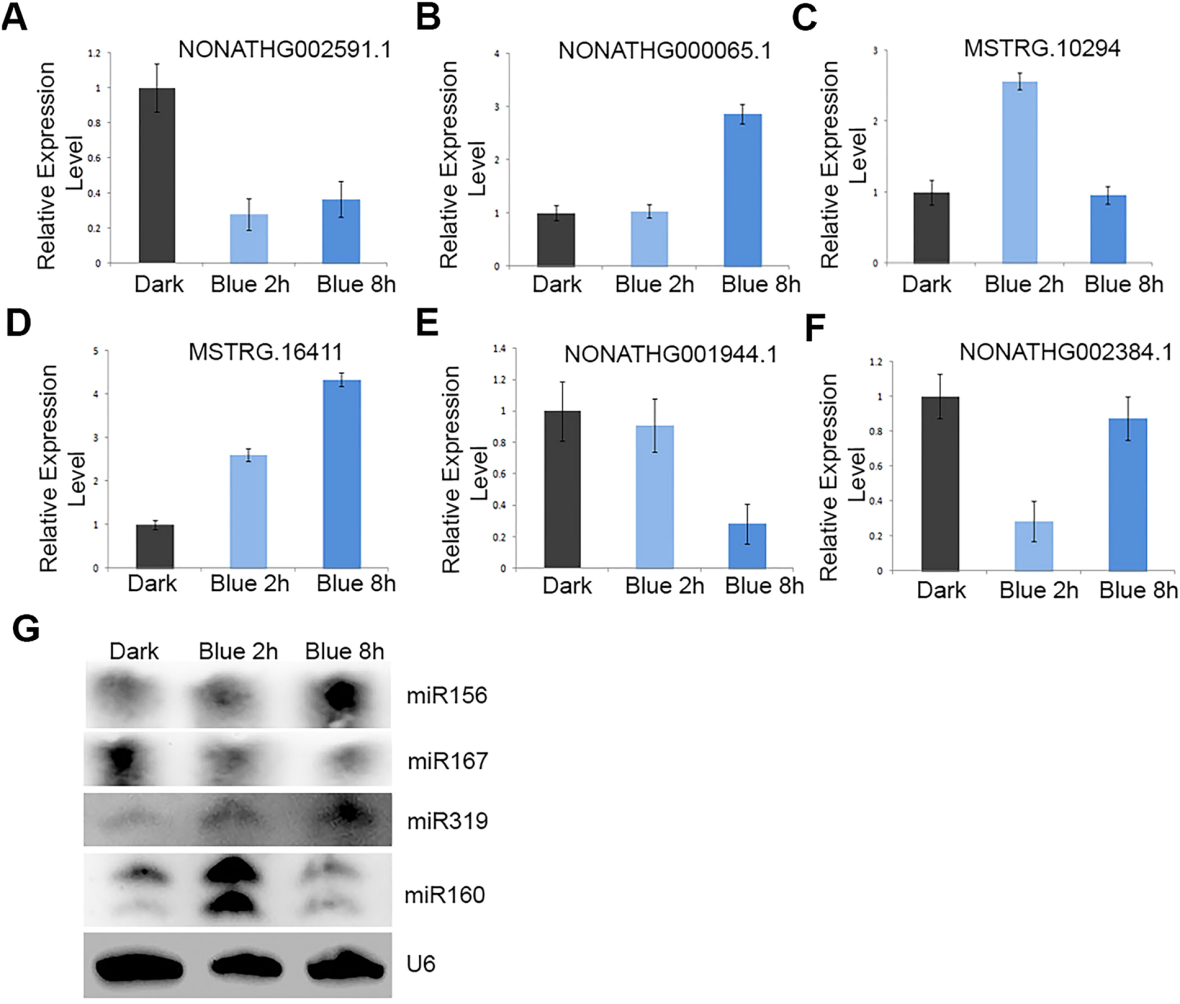
The validation of lncRNA- and miRNA-seq data. **(A**–**F)** The expression patterns of indicated lncRNAs in Col seedlings under blue light treatments for 2 h or 8 h using qRT-PCRs. **(G)** Northern blots show the levels of miRNAs in Col seedlings under blue light treatments for 2 h or 8 h. U6 serves as a loading control.

To validate the expression patterns of lncRNAs under the blue light treatment, we performed qRT-PCR analysis to examine the expressions and sequences of 6 lncRNAs (which include 4 transcripts present in the *Arabidopsis* database (http://www.noncode.org/) and 2 novel transcripts based on our assembly of RNA-seq data) (Fig. 3A–F). The expression patterns of all lncRNAs were found to be consistent with the RNA-seq data. In addition, we also performed Northern blots of 4 miRNAs to validate the results of miRNA sequencing. We found that the expression patterns of the 4 miRNAs were similar to those shown in the miRNA sequencing data (Fig. 3G).

### **Identification of the ceRNA pairs in*Arabidopsis*seedlings under blue light**

**Figure 4:**
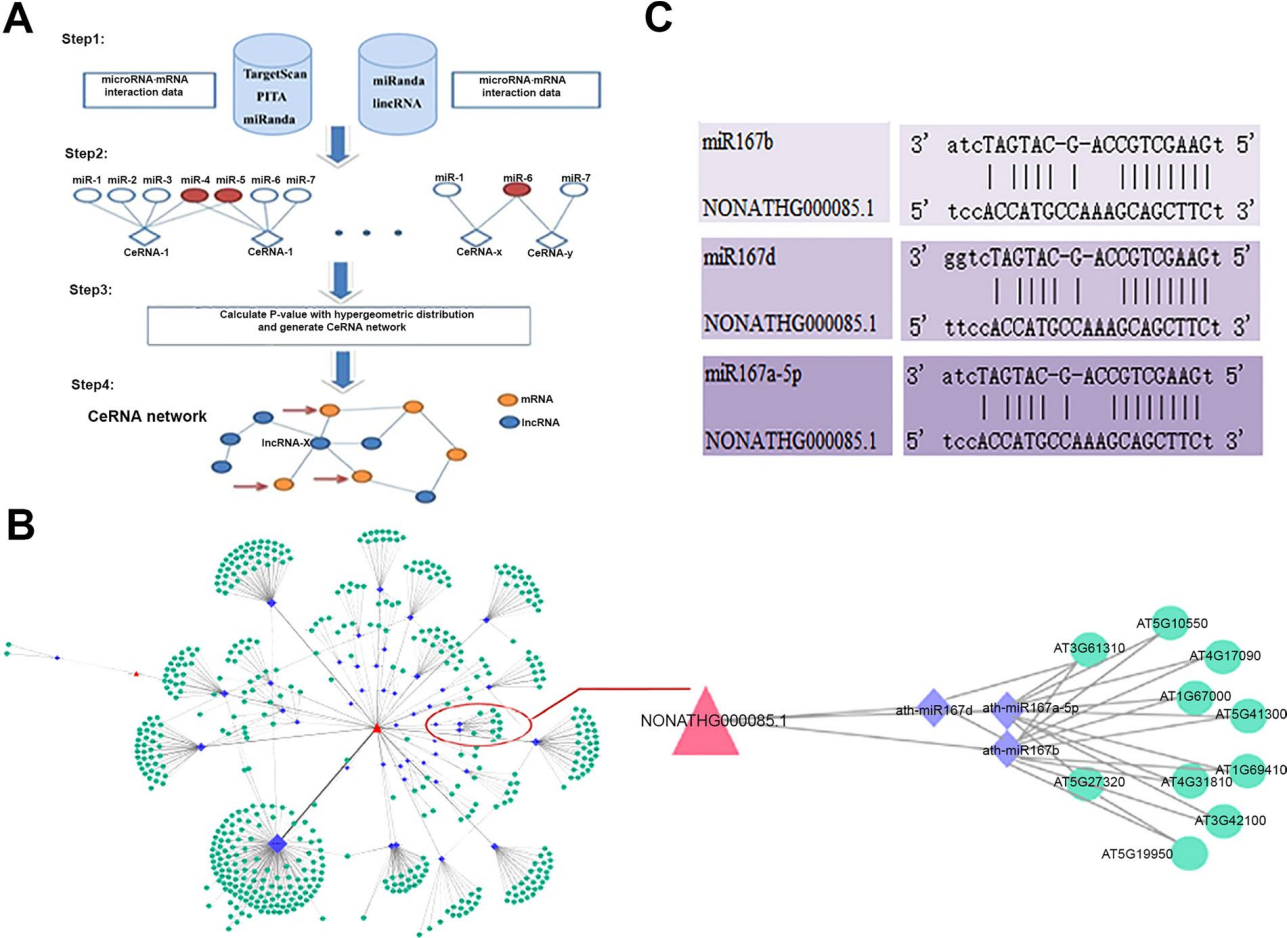
Identification of ceRNAs under blue light treatments. **(A)** CeRNA identification overview. **(B)** CeRNA regulatory networks under blue light treatments. **(C)** The sequence alignment between miRNA167 and lncRNA.

It was known that lncRNA can play a role in sequestering the specific miRNA by acting as a ceRNA in a type of target mimicry to protect the target mRNA in both plants and animals^33–36^. We measured the likelihood of the ceRNAs using StarBase v2.0^37,38^. Using the hypergeometric test^39^, we calculated the *p*-value for each potential ceRNA pair (lncRNA-mRNA)^38^. Our bioinformatics analysis indicated that 180 of the identified lncRNAs have the potential to compete with miRNAs, including several conserved miRNAs such as miR160, miR164, miR167, miR169 and miR172 (Fig. 4A,B, Figs. S2 and S3). We then constructed lncRNA-miRNA-mRNA interaction networks (Figs. S2 and S3). We found that 744 DEGs (differentially expressed genes) and 183 DElncRNAs were targeted by 155 miRNAs (Figs. S2 and S3).

### The role of BLIL1-miR167 pair in blue light-directed plant photomorphogenesis

**Figure 5:**
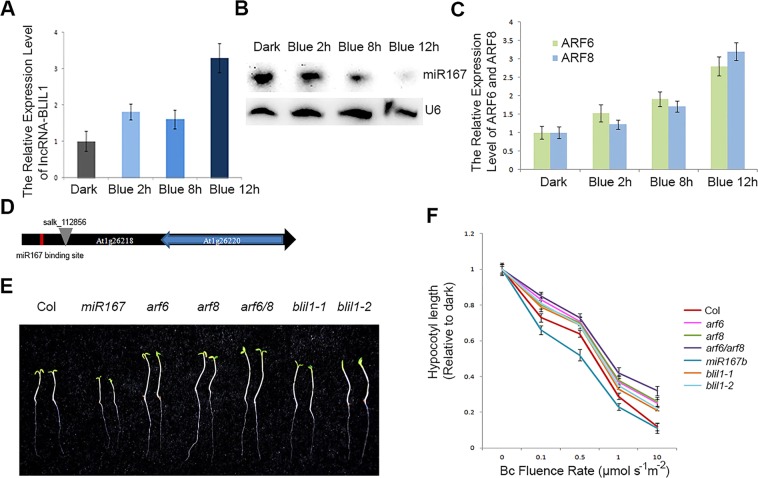
*MiR167* and *BLIL1* play opposite roles in plant photomorphogenesis. **(A)** The relative transcript levels of *BLIL1* in Col seedlings under blue light for 2 h, 8 h and 12 h. **(B)** Northern blots show the levels of *miRNA167* in Col seedlings under blue light for 2 h, 8 h and 12 h. U6 serves as a loading control. **(C)** The expression patterns of *ARF6* and *ARF8* in Col seedlings under blue light treatments for 2 h, 8 h and 12 h. **(D)** Diagram of *blil1-1* mutant. **(E)** Visual phenotypes of Col*, miR167b, arf6, arf8, arf6/8, blil1-1* and *blil1-2* seedlings grown under blue light (10 μmolm^−2^s^−1^) for 4 days. **(F)** Bc fluence rate response curves for hypocotyl lengths of Col*, mir167b, arf6, arf8, arf6/8, blil1-1* and *blil1-2* seedlings grown under blue light (10 μmolm^−2^s^−1^) for 4 days. Data are means ± SEM of 50 plants. All the experiments have been performed for three biological replicates.

We found that miR167 plays a role in modulating hypocotyl elongation^27^, therefore, we focused on the regulatory network of lncRNA-miRNA167-mRNA. The 14 complementary nucleotides of miR167 to BLIL1 are shown in Fig. 4, including 8 bases at the 5′ end of miR167 (Fig. 4B,C). To confirm the expression profiles of BLIL1 and miR167, we performed qRT-PCRs to examine the transcript levels of BLIL1 and miR167-targeted mRNAs under blue light treatments for 2 h, 8 h and 12 h. As shown in Fig. 5A–C, the transcript levels of *BLIL1*, *ARF6* and *ARF8* which are targets of miR167^40^, were induced under blue light treatments. Moreover, Northern blots confirmed that the transcript level of *miR167* is opposite to those of *BLIL1*, *ARF6* and *ARF8* (Fig. 5A–C).

We then investigated the role of the BLIL1-ARF6/8-miR167 regulatory network in photomorphogenesis by examining the blue light-dependent inhibition of hypocotyl elongation. We analyzed the hypocotyl growth of 4-day-old Col and *blil1-1*, *blil1-2*, *arf6/8* and *miR167* mutant seedlings grown under continuous blue light at 10 µmol s^−1^m^−2^ intensity, or continuous darkness. The *blil1-1*, *blil1-2*, *arf6/8* mutants display long hypocotyl phenotypes compared with wild type after treatment with continuous blue light, and *miR167* mutant shows shorter hypocotyl than Col under blue light (Fig. 5D–F). The hypocotyl lengths of these mutants were similar to that of Col grown under dark condition (Fig. S4). These results revealed the competitive relationships between miR167 and BLIL1 or ARF6/8 in regulation of the blue light-dependent photomorphogenesis.

### The role of BLIL1-miR167 pair in response to mannitol stress

**Figure 6:**
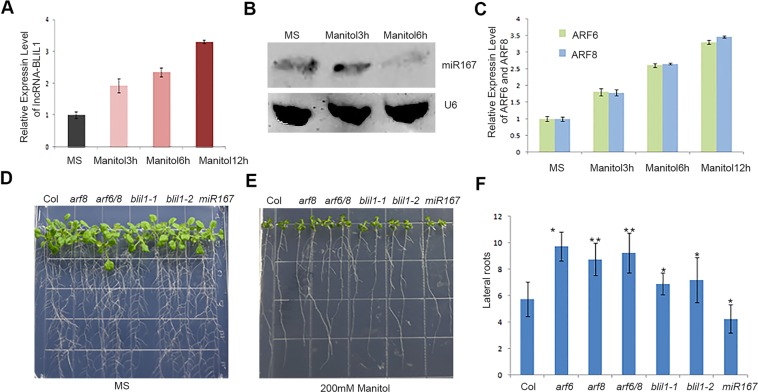
*MiR167* and *BLIL1* play opposite roles under mannitol stress. **(A)** The relative expression levels of *BLIL1* in Col seedlings under 200 mM manitol treatments for 3 h, 6 h and 12 h. **(B)** Northern blots show the levels of *miRNA167* in Col seedlings under 200 mM manitol treatments for 3 h and 6 h. U6 serves as a loading control. **(C)** The relative expression levels of *ARF6* and *ARF8* in Col seedlings under 200 mM manitol treatments for 2 h, 8 h and 12 h. **(D)** and **(E)**, Phenotypes of Col*, miR167b, arf6, arf8, arf6/8, blil1-1* and *blil1-2* plants grown on MS medium (control) **(D)** or MS medium containing 200 mM manitol **(E)**. **(F)** Number of lateral roots of Col*, miR167b, arf6, arf8, arf6/8, blil1-1* and *blil1-2* seedlings on plates containing MS medium with or without 200 mM manitol.

As miR167 was reported to be involved in stress responses^41^, we validated the competitive relationship among BLIL1, ARF6/8 and miR167 under osmotic stresses. We performed qRT-PCRs to examine the induction profile of *BLIL1*, *ARF6/8* and *miR167* under 200 mM mannitol stress. When the seedlings were treated by 200 mM mannitol, the levels of *BLIL1* and *ARF6/8* transcripts increased slightly at 3 h, obviously at 6 h, and reached the maximum at 12 h (Fig. 6A,C), whereas the level of *miR167* decreased obviously at 6 h with no significant difference at 3 h (Fig. 6B).

To further confirm the competitive relationship, we investigated the mannitol tolerances of *blil1-1*, *blil1-2*, *arf6/8* and *miR167* mutant seedlings. Five days after germination on normal MS medium, the seedlings were transferred to MS medium plates containing 200 mM mannitol, and the plates were stood-up. The amounts of lateral roots were measured at the 7th day after transferring. As shown in Fig. 6D–F, the results demonstrated that *blil1-1*, *blil1-2*, *arf6/8* mutant*s* displayed more lateral roots than Col, while *miR167* mutant showed less lateral roots than Col under mannitol stress. As a control, no significant differences were observed among the mutants and Col seedlings under normal MS medium. These results indicated that the mutants of *blil1-1*, *blil1-2*, and *arf6/8* showed reduced sensitivity to mannitol treatment, whereas *miR167* displayed elevated sensitivity to mannitol treatment, suggesting that BLIL1 and ARF*6/8* acts as positive factors, and miR167 as a negative factor in response to mannitol stress.

## Discussion

Blue light plays an important role in plant growth and development^42,43^. Many genes have been known to be involved in blue light signal transduction^44^. The recent discovery of lncRNAs has shown that lncRNAs play roles in many regulatory pathways, such as the regulation of chromatin structure and determination of cell fate^1,2,6–14^. However, up to now, lncRNA has not been shown to be involved in blue light signal transduction. In this study, we identified differentially expressed lncRNAs under blue light compared to the dark condition. We confirmed that an lncRNA (BLIL1) functions as a ceRNA to compete with miRNA167. Further genetics and phenotype analysis of the related mutants supported that BLIL1 is antagonistic to miR167 under blue light and manitol stress.

In this study, we examined blue light-regulated lncRNA and miRNA expressions in 4 d seedlings grown in darkness and then exposed to blue light for 2 h and 8 h. Darkness was used as control instead of white light which is of full spectrum, and contains different wavelengths of lights including blue light. Jiao *et al*. used darkness as the control to analyze the expressions of transcription factors under blue and white light treatments^45^. Wang *et al*. identified long noncoding natural antisense transcripts (lncNATs) from the etiolated seedlings exposing to continuous white light for 1 h or 6 h, and the dark-grown seedlings were used as the control^46^. Darkness was also used as control to examine the transcriptome changes in *cry1cry2* mutation and BLUE-LIGHT INHIBITOR OF CRYPTOCHROMES 1 (BIC1) overexpression lines in response to blue light^47^ and analyze the expression profiles of blue light-grown WT, *cry1cry2*, and CNT1 or CNT1-G283E overexpression seedlings^48^. In addition, we used darkness as control to study miRNA expressions induced under red light treatments for 2 h and 8h^27^.

CeRNAs play important roles in the regulation of miRNA or lncRNA functions. IPS1, a plant lncRNA, is a known functional ceRNA involved in regulation of phosphate assimilation^14^. MiR160 and miR166 for eTMs (endogenous miRNA target mimic) that interact with several lncRNAs were found in *Arabidopsis* and rice^49^. In this study, we constructed a blue light induced ceRNA network of *Arabidopsis*, providing useful resources for studying the blue light signaling. In addition, our ceRNA analysis showed that a single miRNA might target multiple lncRNAs or mRNA (Figs. S2 and S3 in Supporting Information). In animals, lncRNAs were shown to act as ceRNAs to regulate mRNA expression by sponging miRNA in bovine adipocyte differentiation^50^. Nine lncRNAs were proved to act as ceRNAs to affect the expression of lipid metabolic genes by sponging miRNAs in bovine liver^51^. In this work, we focused the intergenic and non-coding sequences of *Arabidopsis thaliana* to predict lncRNAs, and identified 407 ceRNA candidate pairs. Moreover, we verified the competitive function among miR167, BLIL1, and miRNA167 target genes. Many lncRNAs might be functional ceRNAs, which might be important for plant growth and development or stress responses.

LncRNA can regulate the biological activities of eukaryotes through different mechanisms^52^. LncRNA can function as a ceRNA by coordinating with miRNAs, in addition, lncRNAs may regulate gene expression in a *cis*- or *trans*-acting manner^53^. The regulatory roles of lncRNAs in gene expression could be achieved by acting on the adjacent target genes, which was known as the *cis*-acting process of lncRNAs^54^. Some lncRNAs can regulate the translation of mRNA through interfering with the binding of mRNA binding proteins to the mRNA^53^. Cis-Natural Antisense Transcripts (cis-NATs) were known to regulate cognate sense mRNA translation^55^. An example for *trans-*acting lncRNA is *Arabidopsis* hidden treasure 1 (HID1) which associates with the chromatin of the first intron of transcription factor PIF3 and inhibit PIF3 transcription^56^. Several studies also have reported that lncRNAs can regulate the activities of transcription factors^57–59^. It has been known that many regulatory factors involved in light signaling pathways^21,22^, further studies on the connection between lncRNAs and these regulatory factors will provide additional information regarding their detailed roles in light signaling pathway.

## Methods

### Plant materials and growth conditions

*Arabidopsis thaliana* (ecotype Col-0), *miR167b* (CS872594),*arf6* (CS24606), *arf8* (CS24608), *arf6/8* (CS24632), *blil1-1* (SALK_112856) and *blil1-2* (CS65633) mutants were obtained from the Arabidopsis Biological Resource Center (Ohio State University). All plants were grown in soil or Murashige and Skoog (MS) medium at 16 hr light/8 hr dark photoperiod unless specifically indicated otherwise.

### LncRNA and mRNA data analysis

The in-house Perl script was used to process the original reads in fastq format. Clean reads were obtained by removing the reads with adapters and poly-N, and low-quality reads. Q20 (base ratio > 20), q30 (base ratio > 30) and GC content of the obtained clean data were calculated. The high-quality clean data were used for all the subsequent analysis^60^. The *Arabidopsis* reference genome and gene model annotation files were downloaded from ftp://ftp.ensembl.org/pub/release-80. We built an index of the reference genome using Bowtie v2.0.680 and aligned paired-end clean reads to the reference genome using TopHat v2.0.981. The mapped reads of each sample were assembled by Stringtie^61^.

### MiRNA data analysis

Fastx_uutoolkit-0.0.13.2 was used to pre-process the original data by removing low-quality reads, 5′ joint contaminated reads, 3′ spliced reads, and reads with length less than 18 nt or with poly-A. The clean reads were then compared with known ncRNAs by using blast (v2.2.26) in RFAM database (v10.1). Clean reads matching any sequence in the RFAM database was deleted. Then, the proportion of four bases (A/T/G/C) in the clean reads at each location was calculated. The *Arabidopsis* miRNA sequences were downloaded from miRBase (http://www.mirbase.org/), and used to search for the clean reads consistent with the *Arabidopsis* reference genome^60^.

### Identification of the differentially expressed lncRNAs, mRNAs and miRNAs

We used CuffDiff (V2.1.1) to calculate the values of lncRNAs and coding mRNAs for FPKM (the number of fragments per kilo-base of exon per million mapped fragments), and TPM (transcripts per million) fractions of miRNAs. The differential expressions of miRNAs were analyzed by EdgeR. We analyzed differential expressions of lncRNAs, mRNAs and miRNAs by comparing blue light treatment with dark condition. In any pairwise comparisons, the expressions of lncRNAs and mRNAs with corrected *p*-values < 0.05 and absolute fold-change values>2.0, or miRNAs with *p*-values < 0.05, absolute fold-change values>2.0, and miRDeep2 score ≥1, were considered to be differentially expressed^60^.

### Real-time quantitative PCR

The first strand of cDNA is synthesized from total RNA (1 μg) and M-MLV reverse transcriptase (Promega) using random primers and serves as a template for subsequent PCR amplification. Pri-miRNA levels were detected by qRT-PCRs using Bio-Rad CFX Real-Time System. Actin gene was used as the internal control for normalization of DNA templates. Each PCR was repeated at least three times. Data were analyzed by Bio-Rad iCycler iQ Real-Time Detection system. The transcript level was calculated by the relative 2^–ΔΔCt^ method^62^. The primers used for qRT-PCRs are listed in Table S2 supplemental information.

### Small RNA deep sequencing

Small RNAs were isolated from 4-day-old plant seedlings by using the *mir*Vana miRNA Isolation Kit (Ambion, AM1561) and sequenced by high-throughput sequencing of Illumina HiSeq. 2000. Sequencing data of all known miRNAs were hierarchical clustered in an unsupervised manner to analyze the degree of differential expression of small RNA^63^.

### LncRNA and mRNA deep sequencing

LncRNAs and mRNAs were isolated from 4-day-old plant seedlings. Ribosomal RNAs (rRNAs) were removed using TruSeq Stranded Total RNA with Ribo-Zero Plant kit (Illumina, 20020610) and the rRNA-free residue was obtained by ethanol precipitation. The first-strand cDNA was synthesized using a random Hexamer-primer and reverse transcriptase (Invitrogen). The second strand cDNA was synthesized using RNase H (Invitrogen) and DNA polymerase I (New England BioLabs). Subsequently, the sequencing libraries were generated using the rRNA-free RNA with a NEBNext Ultra Directional RNA Library Prep Kit for Illumina (New England Biolabs, MA, USA), and sequenced by high-throughput sequencing of Illumina Hiseq Xten pair-end 150 (PE150).

### Hypocotyl growth analysis

The seedlings were treated with light as described^64^ with some modifications. The seedlings of each line were cultured in the same 100 mm Petri dish for each repeat. The seeds were soaked in 70% ethanol for 5 minutes, and then in 95% ethanol for 5 minutes. Seeds were vernalizated for 3 days in darkness at 4 °C. The culture dishes were placed in white light for 3 hours to induce germination and in the growth chamber at 21 °C for 21 hours. The dishes were then transferred to experimental light conditions. The seedlings were transplanted into blue light (0.1, 0.5, 1 and 10 µmol s^−1^m^−2^) in a growth chamber (Percival Scientific Inc., Perry, Iowa, USA) for 4 days at 21 °C, respectively. Dark control seedlings were kept in darkness. The hypocotyl lengths of at least 30 seedlings were measured after blue light or dark treatments.

### Mannitol stress treatments

Five days after germination on normal MS medium, the wild type and *blil1-1*, *blil1-2*, *arf6/8* and *miR167* mutant seedlings were transferred to MS medium plates with or without 200 mM mannitol, and the plates were stood-up. The phenotypes of seedlings were visualized 7 days after transferring.

### Small RNA gel blot

Small RNA gel blot was performed as previously described^24^. Small RNAs were isolated from 4-day-old plant seedlings by using *mir*Vana miRNA Isolation Kit (Ambion, AM1561). Small RNAs of about three micrograms were fractionated on a 15% polyacrylamide gel containing 8 M urea and then transferred to a nylon transfer membrane (GE Healthcare). Antisense oligonucleotides (Table S2 Supplemental information) were synthesized and biotin probes were labeled at 3′ end. Hybridization was carried out overnight in Ambion (AM8663) at 42 °C. A probe complementary to U6 (5′CATCCTTGCGCAGGGGCCA 3′) was used as a loading control.

## Supplementary information

Supplementary information.

## Data Availability

All necessary data generated or analyzed during the present study are included in this published article, its Supplementary Information files and NCBI (the National Center for Biotechnology Information) with the number PRJNA596364.
