# Author Correction: Genome-wide identification of *Arabidopsis* long noncoding RNAs in response to the blue light

**DOI:** 10.1038/s41598-020-68529-7

**Published:** 2020-07-07

**Authors:** Zhenfei Sun, Kai Huang, Zujing Han, Pan Wang, Yuda Fang

**Affiliations:** 1 Joint Center for Single Cell Biology, School of Agriculture and Biology, Shanghai Jiao Tong University, Shanghai, 200240 China; 2 National Key Laboratory of Plant Molecular Genetics, CAS Center for Excellence in Molecular Plant Sciences, Institute of Plant Physiology and Ecology, Chinese Academy of Sciences, University of Chinese Academy of Sciences, Shanghai, 200032 China; 3 Beijing igeneCode Biotech CO., Ltd, Beijing, 100096 China

Correction to: Scientific Reports 10.1038/s41598-020-63187-1, published online 10 April 2020

This Article contains errors in the Reference list. References 28 and 29 are incorrectly listed as references 30 and 31 respectively. As a result, references 30 and 31 are listed incorrectly as references 28 and 29. The correct references 28–31 are listed below as references 1–4 respectively.

1. Kong, L. et al. Cpc: Assess the Protein-Coding Potential of Transcripts Using Sequence Features and Support Vector Machine. *Nucleic Acids Res.* **35**, W345–W349 (2007).
2. Sun, K. et al. Iseerna: Identification of Long Intergenic Non-Coding Rna Transcripts from Transcriptome Sequencing Data. *BMC Genomics*. **14**(Suppl 2), S7 (2013).
3. Kim, D., Langmead, B. & Salzberg, S.L. Hisat: A Fast Spliced Aligner with Low Memory Requirements. *Nat. Methods*. **12**, 357–360 (2015).
4. Pollier, J., Rombauts, S. & Goossens, A. Analysis of Rna-Seq Data with Tophat and Cufflinks for Genome-Wide Expression Analysis of Jasmonate-Treated Plants and Plant Cultures. *Methods Mol Biol*. **1011**, 305–315 (2013).

