# Supplementary material for: Genome-wide identification of Arabidopsis long noncoding RNAs in response to the blue light
Source: Sci Rep. 2020 Apr 10;10:6229. doi: 10.1038/s41598-020-63187-1 (PMC7148362; doi:10.1038/s41598-020-63187-1)
Supplement: Supplementary file 1 — Supplementary information. [file 41598_2020_63187_MOESM1_ESM.pdf]

Genome-wide identification and functional analysis *Arabidopsis* long noncoding RNAs in response to the blue light

Zhenfei Sun[1,2], Kai Huang[3], Zujing Han[3], Pan Wang[1], Yuda Fang[1,2]

1. Joint Center for Single Cell Biology, School of Agriculture and Biology, Shanghai Jiao Tong University, Shanghai 200240, China
2. National Key Laboratory of Plant Molecular Genetics, CAS Center for Excellence in Molecular Plant Sciences, Institute of Plant Physiology and Ecology, Chinese Academy of Sciences; University of Chinese Academy of Sciences, Shanghai 200032, China
3. Beijing igeneCode Biotech CO., Ltd, Beijing 100096, China

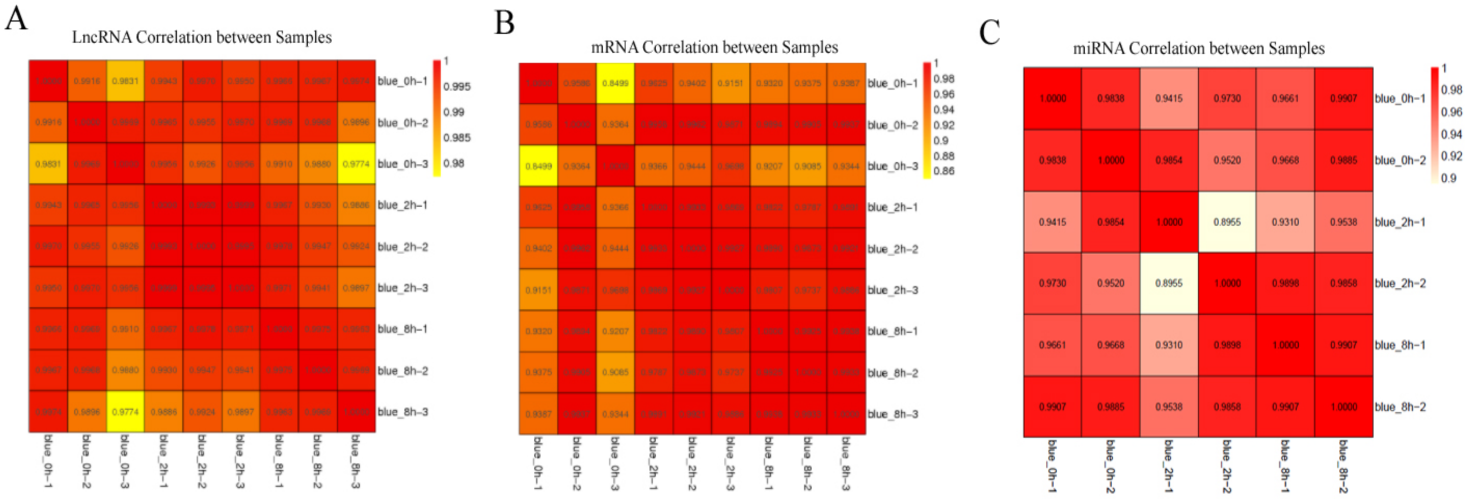

Figure S1. Sample correlation heatmap for lncRNAs**(A)**, mRNAs**(B)** and miRNAs**(C)**.

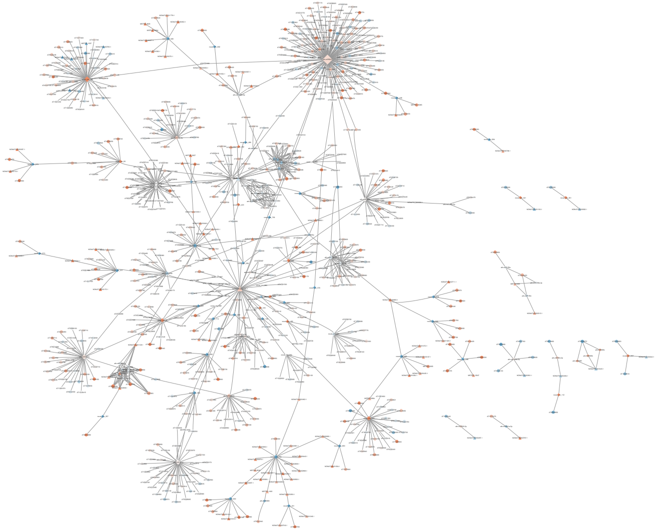

Figure S2. CeRNA network under blue light treatment for 2h.

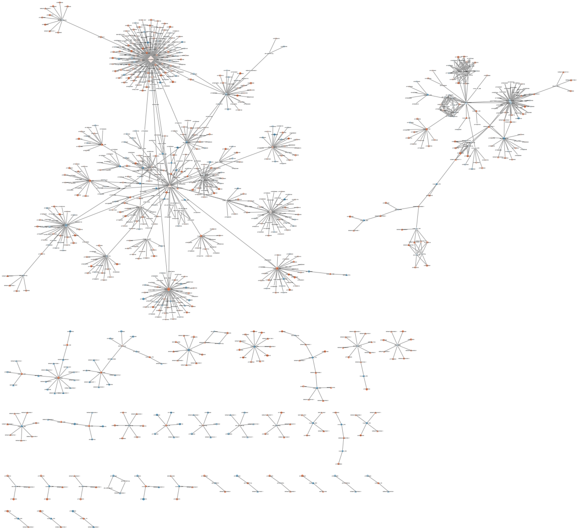

Figure S3. CeRNA network under blue light treatment for 8h.

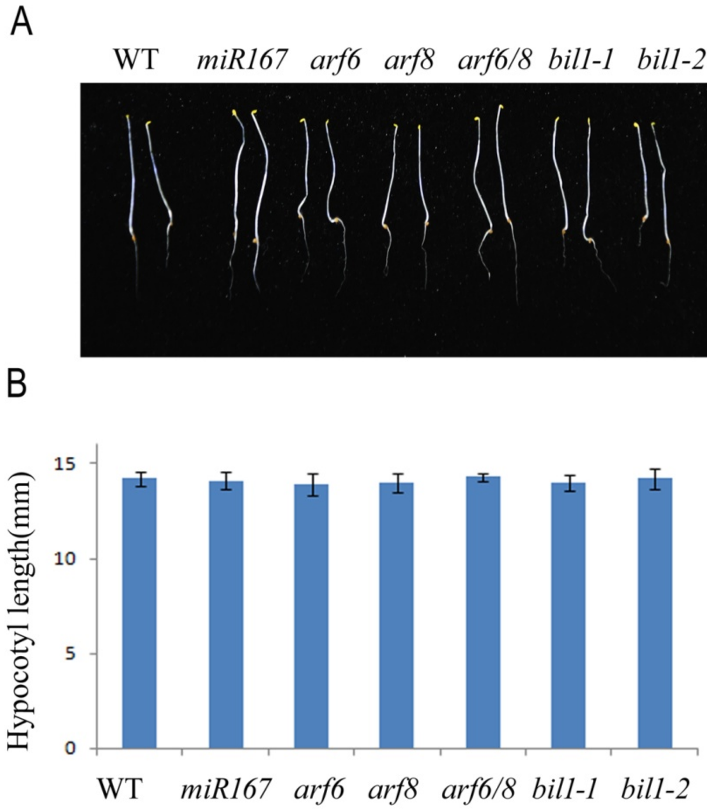

Figure S4. Phenotypes of Col*, mir167b, arf6, arf8, arf6/8, bil1-1 and bil1-2* plants grown in dark.

(A), Visual phenotypes of Col*, miR167b, arf6, arf8, arf6/8, blil1-1 and blil1-2* seedlings grown in dark for 4 days. **(B)**, Hypocotyl lengths of Col*, mir167b, arf6, arf8, arf6/8, blil1-1 and blil1-2* seedlings grown in dark for 4 days. Data are means ± SEM of 50 plants. All the experiments have been performed for three biological replicates.

Figure S5. Full-length blots of Figure 3G

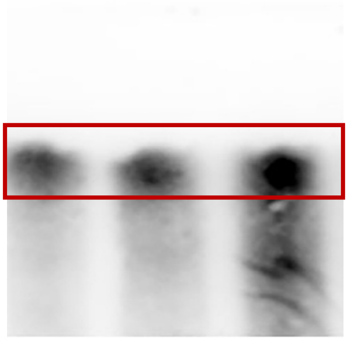

miR156

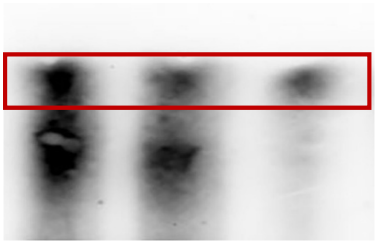

miR167

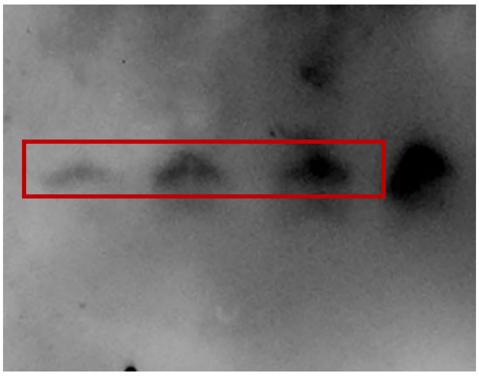

miR319

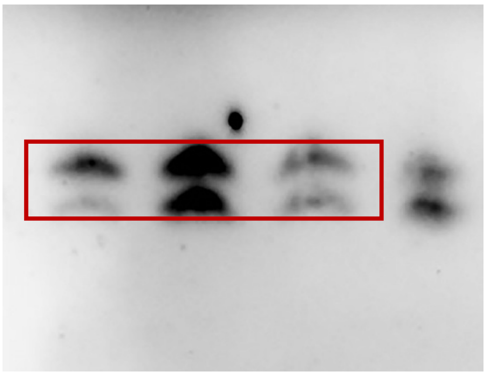

miR160

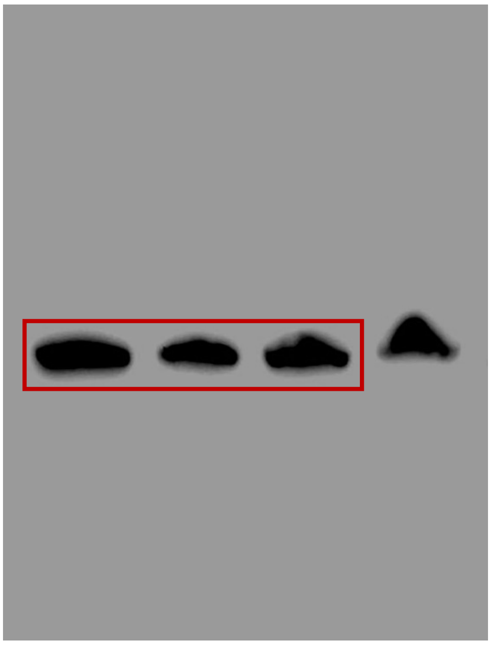

U6

Figure S6. Full-length blots of Figure 5B

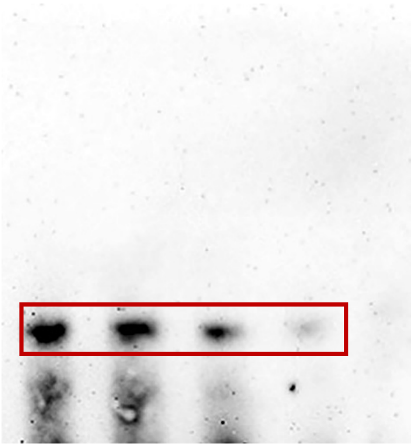

miR167

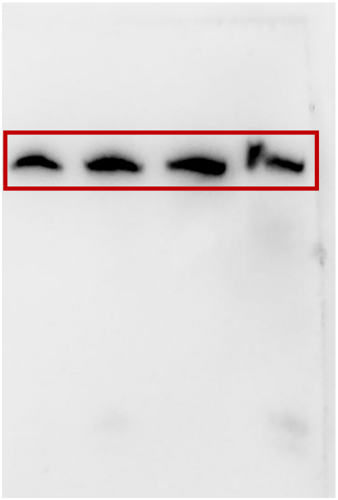

U6

Figure S7. Full-length blots of Figure 6B

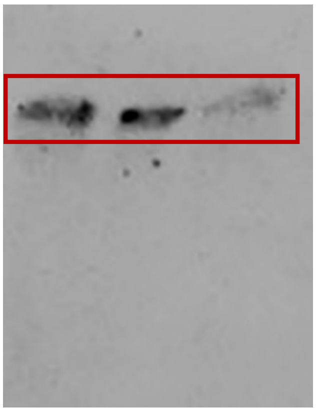

miR167

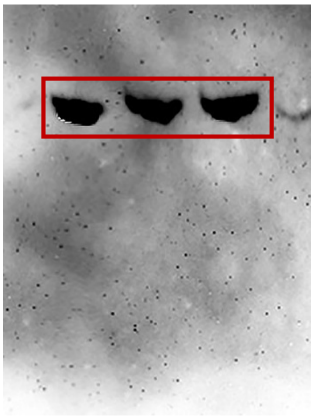

U6

Table S1. mRNA and lncRNA data quality						
Sample	Clean Reads(M)	Clean Bases	Q20(%)	Q30(%)	GC(%)	Read Length(bp)
blue_0h-1	109.5370M	10.9537G	98.63	95.92	53.25	100
blue_0h-2	109.9189M	10.9918G	98.6	95.91	50.15	100
blue_0h-3	109.9875M	10.9987G	98.5	95.65	48.42	100
blue_2h-1	109.8694M	10.9869G	98.52	95.71	50.24	100
blue_2h-2	109.7075M	10.9707G	98.55	95.82	50.05	100
blue_2h-3	109.8973M	10.9897G	98.59	95.92	48.88	100
blue_8h-1	110.0109M	11.0010G	98.56	95.84	49.04	100
blue_8h-2	109.8308M	10.9830G	98.61	95.93	49.73	100
blue_8h-3	110.0180M	11.0018G	98.59	95.95	48.41	100

**Table S2. Oligonucleotide sequences**			
miR156-antisen(3'-biotin)	GTGCTCACTCTCTTCTGTCA	Northern blot	antisense
miR165-antisen(3'-biotin)	GGGGGATGAAGCCTGGTCCGA	Northern blot	antisense
miR167-antisen(3'-biotin)	GATATTGGCGCGGCTCAATCA	Northern blot	antisense
miR319-antisen(3'-biotin)	AGGGAGCTCCCTTCAGTCCAA	Northern blot	antisense
Actine-F	GGTAACATTGTGCTCAGTGGTGG	Real time PCR	sense
Actine-R	AACGACCTTAATCTTCATGCTGC	Real time PCR	antisense
LncRNA-BIL-F	AATGACGCAGCTAATGAG	Real time PCR	sense
LncRNA-BIL-R	TTCCCTTCGGGGTAAAGCC	Real time PCR	antisense
MSTRG.10294-F	GAGGATTTATGCCAAACTACTA	Real time PCR	sense
MSTRG.10294-R	CGAGTCTTGGATTACCATCAGA	Real time PCR	antisense
MSTRG.16411-F	TTTACTCAATTTCTCATTATG	Real time PCR	sense
MSTRG.16411-R	ATCCGTGATGGATATTCCTG	Real time PCR	antisense
NONATHG002591-F	AAAGTCCCTCCGTCTTTGCTTT	Real time PCR	sense
NONATHG002591-R	TCCCACATCGACAAGTTAG	Real time PCR	antisense
NONATHG000065.1-F	TCCCCTTCCATCGATTTAG	Real time PCR	sense
NONATHG000065.1-R	GCTGAATTCTCCTAAAAGCTAT	Real time PCR	antisense
NONATHG001944.1-F	TGGTTGATACAGCTTGAGGA	Real time PCR	sense
NONATHG001944.1-R	TGACGGGGCATCATTCACA	Real time PCR	antisense
NONATHG002384.1-F	CTTGGATCACAATCATCCT	Real time PCR	sense
NONATHG002384.1-R	TCGTCTTTCCATT TTCGTTA	Real time PCR	antisense
